# Lichen planopilaris-like eruption during treatment with tyrosine kinase inhibitor nilotinib*

**DOI:** 10.1590/abd1806-4841.20164724

**Published:** 2016

**Authors:** Juliana Ribeiro Leitão, Neusa Yuriko Sakai Valente, Priscila Kakizaki, Isis Suga Veronez, Mario Cezar Pires

**Affiliations:** 1 Private clinic - São Paulo (SP) - Brazil; 2 Hospital do Servidor Público Estadual de São Paulo (HSPE) - São Paulo (SP), Brazil; 3 Hospital Padre Bento de Guarulhos - Guarulhos (SP), Brazil

**Keywords:** Alopecia, Lichen planus, Protein-tyrosine kinases

## Abstract

Tyrosine kinase inhibitors are effective as a target therapy for malignant neoplasms. Imatinib was the first tyrosine kinase inhibitor used. After its introduction, several other drugs have appeared with a similar mechanism of action, but less prone to causing resistance. Even though these drugs are selective, their toxicity does not exclusively target cancer cells, and skin toxicity is the most common non-hematologic adverse effect. We report an eruption similar to lichen planopilaris that developed during therapy with nilotinib, a second generation tyrosine kinase inhibitor, in a patient with chronic myeloid leukemia resistant to imatinib. In a literature review, we found only one report of non-scarring alopecia due to the use of nilotinib.

## INTRODUCTION

Chemotherapy with tyrosine kinase inhibitors (TKIs) is a major advance in medicine because they target molecules and signaling pathways that are typical of cancerous processes. The treatment allows for greater therapeutic efficacy and increased patient survival. Despite their selective molecular inhibition, not only do these drugs affect tumor cells, several side effects may occur as well. Among them, adverse skin reactions are the most commonly seen.

Nilotinib, a second generation TKI, was approved in 2007 for treatment of Philadelphia chromosome-positive chronic myeloid leukemia (CML). Given its recent therapeutic use, few publications about its side effects are available. In a literature review, we found only one case of non-scarring alopecia due to the use of nilotinib.^1^ This report is, therefore, the first to describe an eruption similar to lichen planopilaris associated with the use of nilotinib in a patient presenting with CML resistant to treatment.

## CASE REPORT

**Figure 1:**
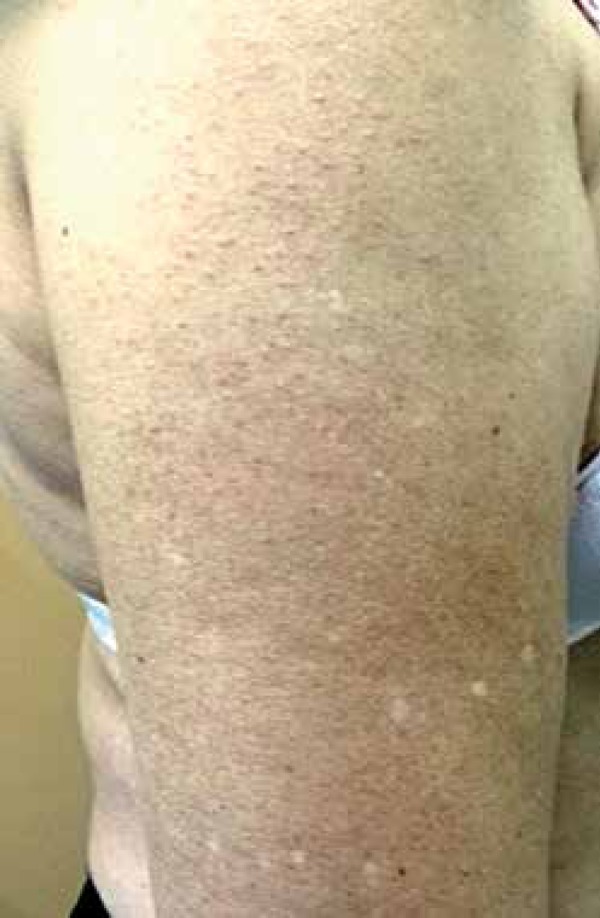
Erythematous papules centered on hair follicles and hair thinning on the right upper limb

**Figure 2:**
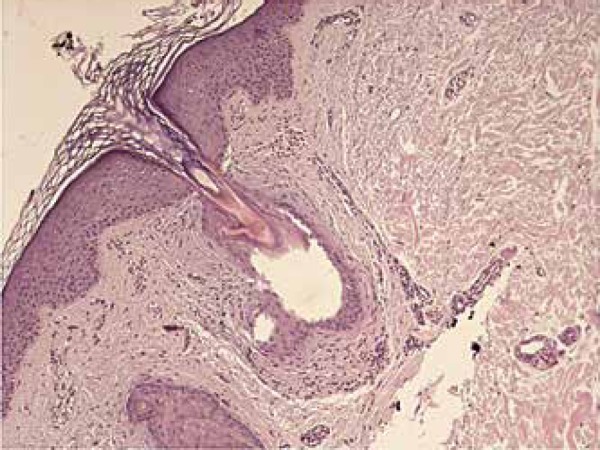
Histopathological aspect similar to lichen planopilaris of a keratotic papule: hair follicle showing fibrosis around the atrophic isthmus and mild lymphocytic infiltrate (200x)

**Figure 3:**
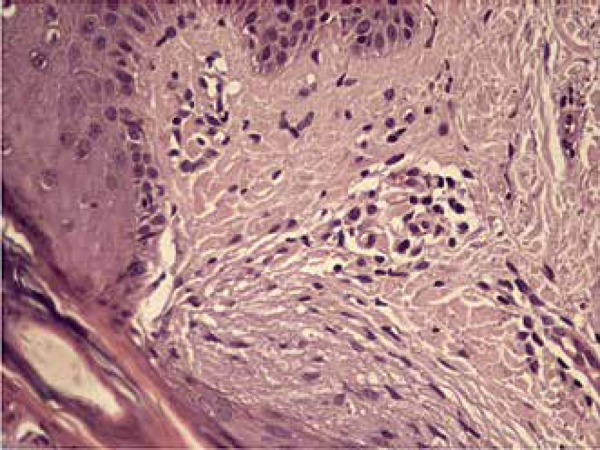
Histopathological aspect similar to lichen planopilaris of a keratotic papule: atrophic isthmus, fibrosis and chronic inflammation of perifollicular vessels (400x)

We report on a 55-year-old female patient diagnosed with CML in 2004 and treated with imatinib (600 mg/daily) from 2007 to 2010. Due to a poor hematologic response, her medication was changed to nilotinib (400 mg/daily). Six weeks after the patient started the new therapy, she showed diffuse itchy skin associated with follicular erythematous papules, hair thinning, and alopecia, especially on the extensor surface of her upper limbs (Figure 1). A biopsy of her upper right limb lesion was performed and it showed a hair follicle with interstitial mucin deposition around the atrophic isthmus and mild perivascular lymphocytic inflammatory infiltrate. The diagnosis of perifollicular fibrosis was discarded. These findings are consistent with lichen planopilaris (Figures 2 and 3). Since the patient could adequately control CML through the use of nilotinib, the medication was not withdrawn and moisturizer and topical corticoids were prescribed to relieve the symptoms.

## DISCUSSION

Nilotinib is an oral chemotherapy drug derived from aminopyridine. It inhibits several tyrosine kinase-like targets, preferably BCR-ABL protein, but also c-kit and the platelet-derived growth factor receptors (PDGFR). It is a second generation TKI and despite its structural similarity with imatinib, it is 30 times more potent because of its higher affinity with and competitiveness against BCRABL. The drug may also be prescribed for patients who are resistant to imatinib.^2,3^ Skin reactions are the most common non-hematologic adverse effect caused by TKIs and they appear to be dose-dependent. In a phase-1 study involving 119 patients with leukemia resistant to imatinib, the most common skin reactions were pruritus (17%-20%), rash (10%-17%), xerosis cutis (13%-17%), alopecia (6%), and Sweet's syndrome (one case). Clinically, rash presents as pruritic perifollicular hyperkeratotic erythematous papules that can affect any part of the body, but it mostly occurs on the trunk and upper limbs.^4,5^ Our patient developed a follicular reaction six weeks after the introduction of a new drug. Because it was not possible to suspend the medication and subsequently reintroduce it to confirm the cause-effect association, a probable causal correlation was established based on the relevant chronological relationship observed. Reports of similar cases in the literature after the use of other targeted therapies also helped with the diagnosis. Follicular rash and alopecia have been described with the use of sunitinib associated with sorafenib. These drugs show affinity for PDGFR fusion proteins and c-kit.^5^ The sharing of therapeutic targets with nilotinib suggests that similar mechanisms of action are responsible for the follicular liquenoid reaction observed in our study. Dermatologists must be able to recognize and establish the diagnosis of these adverse skin reactions to appropriately manage them and relieve their symptoms. These actions will improve patients' quality of life and promote better adherence to chemotherapy, which requires long-term treatment.

## References

[1] Hansen T, Little AJ, Miller JJ, Ioffreda MD (2013). A case of Inflammatory Nonscarring Alopecia Associated With the
Tyrosine Kinase Inhibitor Nilotinib. JAMA Dermatol.

[2] Robert C, Soria JC, Spatz A, Le Cesne A, Malka D, Pautier P (2005). Cutaneous side-effects of kinase inhibitors and blocking
antibodies. Lancet Oncol.

[3] Amitay-Laish I, Stemmer SM, Lacouture ME (2011). Adverse cutaneous reactions secondary to tyrosine kinase
inhibitors including imatinib mesylate,nilotinib and
dasatinib. Dermatol Ther.

[4] Kantarjian H, Giles F, Wunderle L, Bhalla K, O'Brien S, Wassmann B (2006). Nilotinib in imatinibresistant CML and Philadelphia
chromosome-positive acute lymphocytic leukemia. N Engl J Med.

[5] Lee WJ, Lee JL, Chang SE, Lee MW, Kang YK, Choi JH (2009). Cutaneous adverse effects in patients treated with multitargeted
kinase inhibitors sorafenib and sunitib. Br J Dermatol.

